# An empirical study of using radiology reports and images to improve intensive care unit mortality prediction

**DOI:** 10.1093/jamiaopen/ooae137

**Published:** 2025-02-20

**Authors:** Mingquan Lin, Song Wang, Ying Ding, Lihui Zhao, Fei Wang, Yifan Peng

**Affiliations:** Department of Population Health Sciences, Weill Cornell Medicine, New York, NY 10022, United States; Department of Surgery, University of Minnesota, Minneapolis, MN 55455, United States; Cockrell School of Engineering, The University of Texas at Austin, Austin, TX 78712, United States; School of Information, The University of Texas at Austin, Austin, TX 78712, United States; Department of Preventive Medicine, Feinberg School of Medicine, Northwestern University, Chicago, IL 60611, United States; Department of Population Health Sciences, Weill Cornell Medicine, New York, NY 10022, United States; Department of Population Health Sciences, Weill Cornell Medicine, New York, NY 10022, United States

**Keywords:** mortality prediction, deep learning, multimodal fusion

## Abstract

**Objectives:**

The predictive intensive care unit (ICU) scoring system is crucial for predicting patient outcomes, particularly mortality. Traditional scoring systems rely mainly on structured clinical data from electronic health records, which can overlook important clinical information in narratives and images.

**Materials and Methods:**

In this work, we build a deep learning-based survival prediction model that utilizes multimodality data for ICU mortality prediction. Four sets of features are investigated: (1) physiological measurements of Simplified Acute Physiology Score (SAPS) II, (2) common thorax diseases predefined by radiologists, (3) bidirectional encoder representations from transformers-based text representations, and (4) chest X-ray image features. The model was evaluated using the Medical Information Mart for Intensive Care IV dataset.

**Results:**

Our model achieves an average C-index of 0.7829 (95% CI, 0.7620-0.8038), surpassing the baseline using only SAPS-II features, which had a C-index of 0.7470 (95% CI: 0.7263-0.7676). Ablation studies further demonstrate the contributions of incorporating predefined labels (2.00% improvement), text features (2.44% improvement), and image features (2.82% improvement).

**Discussion and Conclusion:**

The deep learning model demonstrated superior performance to traditional machine learning methods under the same feature fusion setting for ICU mortality prediction. This study highlights the potential of integrating multimodal data into deep learning models to enhance the accuracy of ICU mortality prediction.

## Introduction

Predictive ICU scoring systems are essential for measuring disease severity and predicting patient outcomes, especially mortality, in the intensive care unit (ICU).^1^ These systems, such as the Acute Physiology and Chronic Health Evaluation,^2^ Simplified Acute Physiology Score (SAPS) II,^3^ and Mortality Probability Model,^4^ rely mainly on structured clinical data, including demographics, vital signs, and lab tests recorded in electronic health records (EHRs).

Recent advances in machine learning have shown promise in improving ICU mortality prediction.^5–8^ However, most studies have focused on structured data, potentially overlooking critical information in narratives and images.^9^^,^^10^ To overcome this issue, many studies focus on mining unstructured clinical notes for patient mortality prediction.^11–13^ However, most of these works were not compared with the current scoring system, making it challenging to compare these models fairly.

Moreover, the practice of modern medicine usually relies on multimodal information. Consequently, many feature fusion strategies were proposed to enhance the performance of prediction algorithms, such as early fusion, late fusion, and joint fusion.^14^ Early fusion combines multimodal features into a single vector by concatenating or averaging.^15–17^ Late fusion combines the predictions of multiple models to make the final decision.^18–20^ Joint fusion combines the features from the intermediate layer of the neural network with the features of other modalities. The loss during training will propagate back to the feature extraction neural network, thereby creating a better feature representation through training iterations.^14^^,^^21–23^ Despite these encouraging findings, we note that most competitive approaches studied the classification tasks. Thus, the integration of text and images in the survival analysis framework remains an important yet, to date, insufficiently studied problem.

Our study aims to address these limitations by incorporating natural language processing (NLP) and medical image analysis to extract hidden features from radiology reports and chest X-rays, which may not be captured in the structured EHR.^24^ We investigate deep learning models for superior ICU mortality prediction compared to traditional machine learning models.^25^ Specifically, we first build the clinical prediction models to predict ICU mortality using the SAPS-II risk factors such as demographics, vital signs, and lab tests. These measurements were obtained in the first 24 hours of ICU admission. We then enrich the model with multimodal features extracted from radiology reports and chest X-rays. The radiology imaging and reading were studied in the first 24 hours. We hypothesize that integrating free texts and images with clinical measurements will improve prediction accuracy. Experiments on the MIMIC-IV dataset^26^ demonstrate that our multimodal models significantly outperform unimodal models.

Our framework offers several important strengths: it effectively fuses multimodal data for ICU mortality prediction, outperforms existing clinical standards (SAPS-II), and is publicly available for reproduction by others.

## Materials and methods

### Dataset

**Figure 1.**
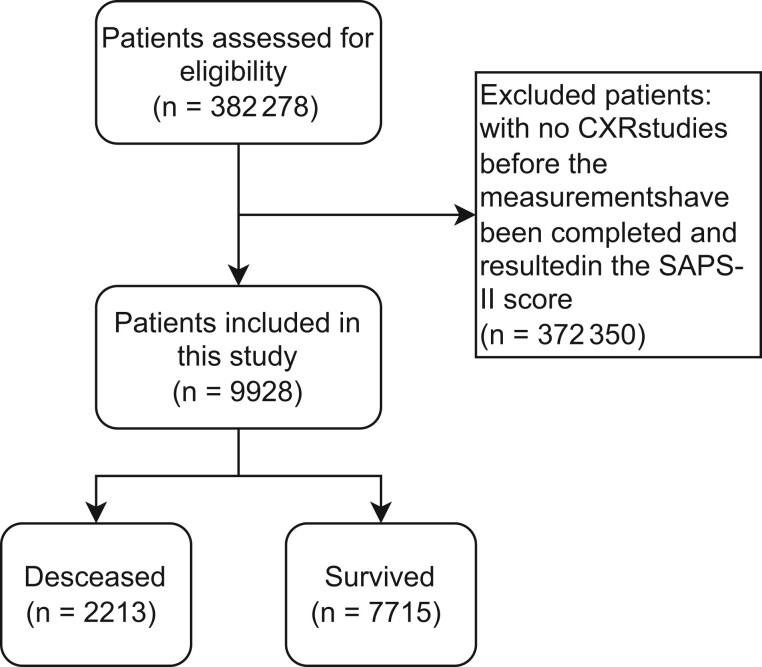
Creation of the dataset.

We used the Medical Information Mart for Intensive Care IV (MIMIC-IV) dataset to evaluate the proposed model.^26^ Medical Information Mart for Intensive Care IV was a deidentified clinical database composed of 382 278 patients admitted to the ICUs at Beth Israel Deaconess Medical Center. Of those, we excluded patients who had no chest x-ray (CXR) studies before the measurements were completed and resulted in the SAPS-II score. Therefore, a total of 9928 patients were included in this study (Figure 1). Out of these patients, 2213 patients (22%) were deceased in the ICU. Table S1 lists the information on the ICU admission group studied in this work. Details of the SAPS-II can be found in Table S2.

### Task

We first formulated the survival analysis task, which predicted a patient’s survival probability in the ICU as a function of their features. We had n patients $\left(x_{i},y_{i},\delta_{i}\right)$. Each patient record consisted of d potential covariants $x_{i}\in R^{d}$, and the time $T_{i}$ when the death occurred or the time $C_{i}$ of censoring. Since death and censoring were mutually exclusive, we use the indicator $\delta_{i}\in \{0,1\}$ and the observed survival time $y_{i}$, defined as below:


$$y_{i}=\min(T_{i},C_{i})=\left\{\begin{array}{cc} T_{i} & \text{if}\;\delta_{i}=1\\ C_{i} & \text{if}\;\delta_{i}=0 \end{array}\right.$$

The goal was to estimate the survival probability $S_{i}(t)=Pr_{i}(T>t)$ of a patient who was not dead beyond time t.

In this study, we used one of the most popular survival analysis models, the Cox model,^27^ where the survival function was assumed to be


$$S_{i}(t|x_{i})=S_{0}(t{)}^{e^{\psi (x_{i})}}$$

In this model, $S_{0}(t)$ was the baseline survival function that describes the risk for individuals with $x_{i}=0$, and $\psi (x_{i})=x_{i}\beta$ was the relative risk based on the covariants. Note that $S_{0}(t)$ was shared by all patients at time t. It was not associated with any individual covariants. The effect of the covariate values $x_{i}$ on the survival function was to raise it to a power given by the relative risk.

In the Cox model, $\psi (x_{i})$ had the form of a linear function, but we also extended it to a nonlinear risk function of a neural network, called the DeepSurv-based model. The DeepSurv-based model had 3 steps: feature extraction, multimodal feature fusion, and survival analysis. The main difference between our model and the DeepSurv model^28^ was that our deep network performs multimodal feature fusion. When only a single modality was input, our model was equivalent to the DeepSurv model. The details of the neural network via feature fusion are described in the next section.

### Neural network via feature fusion

The practices of physicians relied heavily on the synthesis of data from multiple sources. This includes, but was not limited to, structured laboratory data, unstructured text data, and imaging pixel data. Therefore, automated predictive models that successfully utilize multimodal data may lead to better performance.

**Figure 2.**
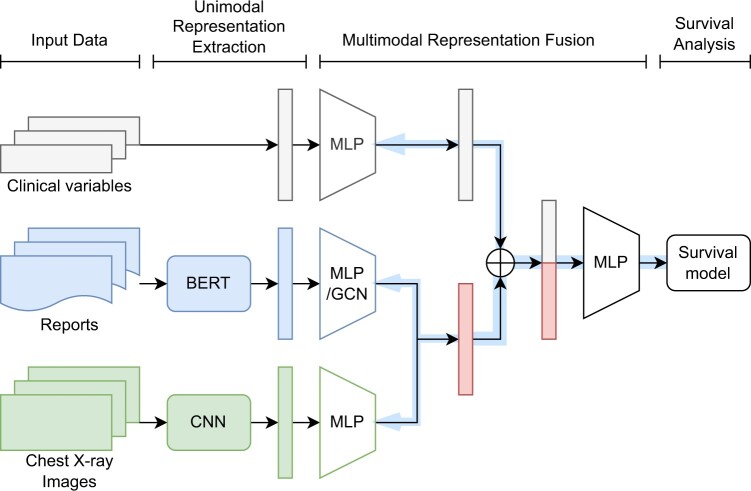
Multimodal feature fusion network.

In this paper, we expanded $\psi (x_{i})$ by introducing a deep neural network with the fusion features from multiple sources: SAPS-II risk factors $x_{\mathrm{saps}}$, text features $x_{\mathrm{text}}$, and imaging features $x_{\mathrm{img}}$, as shown in Figure 2. The extracted text features $x_{\mathrm{text}}$ and image features $x_{\mathrm{img}}$ were passed to 2 separate multilayer perceptron (MLP) modules where the output dimensions are equal. We then used the 2 hidden features by elementwise averaging. Finally, we concatenated it to $x_{\mathrm{saps}}$.


$$x_{i}=\mathrm{Avg}({\text{DNN}}_{\mathrm{img}}(x_{\mathrm{img}}),{\text{DNN}}_{\mathrm{text}}(x_{\mathrm{text}}))⊕x_{\mathrm{saps}}$$

Regarding fusion strategy, our approach was similar to “early fusion,” which refers to combining features from multiple input modalities into one feature vector before feeding it into the survival model.^14^ The difference was that our loss was propagated back to the DNNs during training, thus creating better feature selections for each training iteration. In addition, our approach was not “joint fusion” because the parameters of the features were not updated during the training iteration.

### Feature extraction

Our feature extraction includes 3 components: SAPS-II score and risk factors extraction, text feature extraction, and image feature extraction.

#### SAPS-II score and risk factors

Simplified Acute Physiology Score-II was designed to measure the disease severity of patients aged 18 or more admitted to ICU.^3^ Twenty-four hours after admission to the ICU, the measurements were completed, and the result was an integer point score between 0 and 163. The score was calculated from 15 routine physiological measurements, including information about previous health status and some information obtained at admission. These measurements were: age, heart rate, blood pressure, temperature, PaO_2_/FiO_2_, blood urea nitrogen, urine output, sodium, potassium, bicarbonate, bilirubin, white blood count, Glasgow Coma Scale, chronic disease, and admission type.

#### Text features

In this work, we investigate 3 sets of text features.

##### Common thorax diseases from radiology reports

The first set of features consisted of 13 predefined diseases commonly found in radiology reports (atelectasis, cardiomegaly, consolidation, edema, enlarged cardiomediastinum, fracture, lung lesion, lung opacity, pleural effusion, pleural other, pneumonia, pneumothorax, support devices) and normal,^29–31^ as shown in Figure 2. These labels were extracted from radiology reports using NegBio^32^ and could be obtained from the MIMIC-CXR website (https://physionet.org/content/mimic-cxr-jpg/2.0.0/).

##### Transformer-based features

The second set of features were text embeddings extracted by the bidirectional encoder representations from transformers (BERT) model, which benefited from pretraining on large-scale biomedical and clinical text corpora. Clinical texts were challenging to use in survival analysis due to their unstructured nature. The predefined lung disease labels may not capture all relevant textual information, as they were limited in scope. In this work, we utilized BERT-based hidden layer representations as text features. For a given input report that contains m tokens, the BERT model produced a d-dimension embedding vector for each token, resulting in an m×d representation vector of the report in the latent space. We then applied average pooling over the token embeddings from the last layer of the BERT model to obtain an aggregate latent representation of the report.

##### Graph convolutional neural network-based features

We built a graph convolutional network (GCN) to model the inner correlations among radiology concepts. The graph was manually defined by domain experts (Figure 1 in Irvin et al^30^). Disease findings were defined as nodes and correlated findings were connected to influence each other during graph propagation. We took the m×d hidden representation vectors from the last layer of the BERT model. To initialize GCN node features, we applied a 1-dimension convolution over the text features with the kernel size k and the number of output channels equal to the number of graph nodes. In this way, the graph nodes were initialized by aggregating the hidden features of all the tokens in the report.

The GCN updated its node representations by message passing. We first calculated $\hat{A}=D^{-1/2}\tilde{A}D^{-1/2}$ in a preprocessing step. $\tilde{A}$ = A + $I_{N}$ was the adjacency matrix with added self-connections, where A was the graph adjacency matrix, $I_{N}$ was the N-dimension identity matrix, $D=\text{diag}\sum_{j}A_{ij}$ was the diagonal node degree matrix. Then, based on the study of Kipf and Welling,^33^ the graph convolution could be expressed as follows:


$$H^{1}=\mathrm{ReLu}(\hat{A}H^{0}W^{0}+b^{0})Z=\mathrm{softmax}(\hat{A}H^{1}W^{1}+b^{1})$$

where $H^{l}$ are the states in the *l*th layer, with $H^{0}$ initialized using the aggregate report text hidden features, and $W^{l}$ is a trainable layer-specific weights matrix.

#### Image features

For image feature extraction, we used ChexNet, a DenseNet-121 model pretrained on the CheXpert dataset.^30^^,^^34^^,^^35^ For each input image, we extracted the image features of dimension $d_{\mathrm{img}}$ from the global average pooling layer of DenseNet-121.2.4 Study population and patient selection.

### Evaluation metrics

To assess the accuracy of our models, we used the C-index, defined as follows:


$$\begin{array}{c} L_{s}=\frac{\sum_{i,j}I(T_{i}\geq T_{j})\cdot I(R_{i}\leq R_{j})}{\sum_{i,j}I(T_{i}\geq T_{j})\cdot d_{j}}, \end{array}$$

where $I(c)=\left\{\begin{array}{cc} 1 & \text{if}\;c\;\text{is}\;\text{true}\\ 0 & \text{otherwise} \end{array}\right.$, $d_{j}=\left\{\begin{array}{cc} 1 & \text{if}\;T_{j}\;\text{exist}\\ 0 & \text{otherwise} \end{array}\right.$, j∈{1,2, …,N}, and j>i. N is the number of samples. Intuitively, the C-index measures the extent to which the model can assign logical risk scores. An individual with a shorter time-to-event T should have a higher risk score R than those with a longer time-to-event. C-index assigned a random model of 0.5 and a perfect model of 1.

### Implementation and experimental settings

We performed a grid search to find the optimal hyperparameters based on the metrics and used them for all configurations. The MLP layer for SAPS-II risk factors took an input of 15 dimensions and fully connected to 15 output dimensions. The MLP layer for label features fully connected the 14-dimension inputs to the 14-dimension outputs. The MLP layer for report text features fully connected the 768-dimension inputs to the 32-dimension outputs, and the MLP layer for chest X-ray image features fully connected the 1024-dimension inputs to the 32-dimension outputs.

We used 200 bootstrap samples to obtain a distribution of the C-index and report the 95% CI. For each bootstrap experiment, we sampled n patients with replacements from the whole set of n patients. We then split the sampled set into training (70%), validation (10%), and test (20%) sets. We iterate the training process for 250 epochs with batch size 72 and early stop if the validation loss does not decrease. The dropout rate was 0.5. The learning rate was 0.001 with an Adam optimizer.^36^

We obtained the SAPS-II scores using the scripts in the MIMIC-IV repository (https://github.com/MIT-LCP/mimic-iv). The text embeddings are extracted using BlueBERT,^37^ which was pretrained on the PubMed abstracts and MIMIC-III notes. We used pycox (https://github.com/havakv/pycox), scikit-survival,^38^ and PyTorch to implement the framework. Intel Core i9-9960X 16 cores processor and NVIDIA Quadro RTX 5000 GPU were used in this work. The SAPS-II score was commonly used in ICU mortality prediction and could be directly obtained from the MIMIC-IV website for the MIMIC-IV dataset.

## Results

### Comparison of ICU scoring models and our models with 4 different feature settings

We first compare the baseline ICU scoring model and our models with 4 different feature settings. The SAPS-II score is an integer point score between 0 and 163 directly obtained from the MIMIC-IV website. The SAPS-II risk factors model is trained using the 15 routine physiological measurements. The SAPS-II risk factors + GCN features model is enriched with the GCN-based features. The SAPS-II risk factors + Image features model is enriched with chest X-ray image features. The multimodal features model is trained using SAPS-II risk factors, text features, and chest X-ray image features using early average fusion.

Table 1 shows that the ICU scoring model achieves an average C-index of 0.7470 (95% CI, 0.7263-0.7676). The mean C-index of our model with SAPS-II risk factors achieves 0.7545 (0.7240-0.7849), which brings 0.75% improvements to the ICU scoring baseline model. When combining the SAPS-II risk factors with GCN-based text features and image features, the models obtain the average C-index of 0.7720 (0.7517-0.7923) and 0.7752 (0.7518-0.7985), respectively, yielding increases of 2.50% and 2.82%. Using the multimodal features, the performance of the model can further be boosted. We obtain the average C-index of 0.7829 (0.7620-0.8038), resulting in an improvement of 3.60% over the ICU scoring model. Using early average fusion, we also train the multimodal features model with SAPS-II risk factors combined with GCN features and chest X-ray image features. The average C-index is 0.7805 (0.7570-0.8040), which is slightly lower than the proposed multimodal features model.

**Table 1.** C-index comparison of the models using different sets of features.

Model	C-index (95% CI)
SAPS-II scores (ICU scoring baseline)	0.7470 (0.7263-0.7676)
SAPS-II risk factors	0.7545 (0.7240-0.7849)
SAPS-II risk factors + GCN features	0.7720 (0.7517-0.7923)
SAPS-II risk factors + Image features	0.7752 (0.7518-0.7985)
Multimodal features	0.7829 (0.7620-0.8038)

Abbreviations: GCN, graph convolutional network; ICU, intensive care unit; SAPS-II, Simplified Acute Physiology Score II.

**Figure 3.**
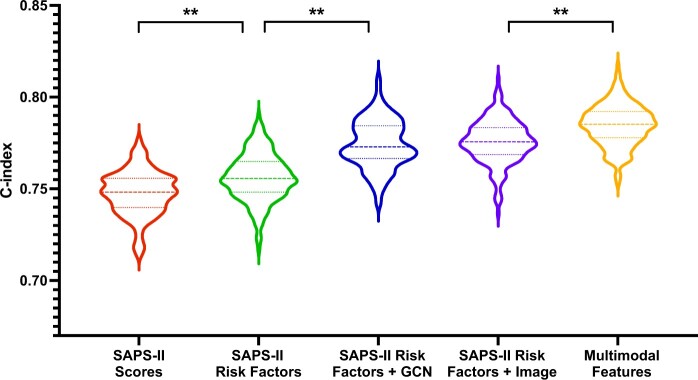
C-index comparisons of the models using different sets of features. **P < .01.

Figure 3 shows more details on bootstrapping. The violin shape reflects the distribution of the C-index: the thicker, the higher the frequency. We find that the average C-index associated with the multimodal features model is statistically higher than the other 4 settings.

**Figure 4.**
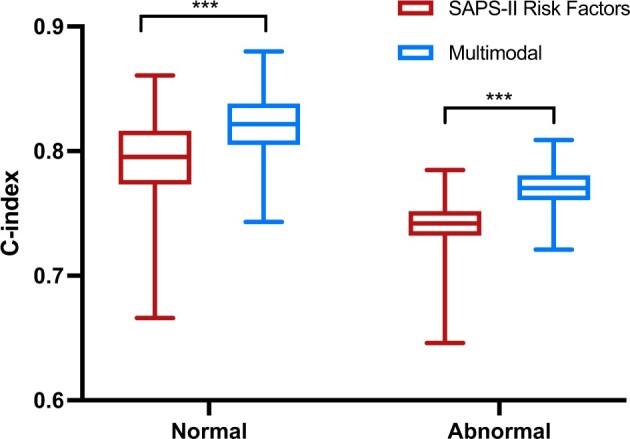
The C-index results of the models trained on the entire dataset and tested on normal patients or patients with chest X-ray abnormalities. ***P < .001.

**Figure 5.**
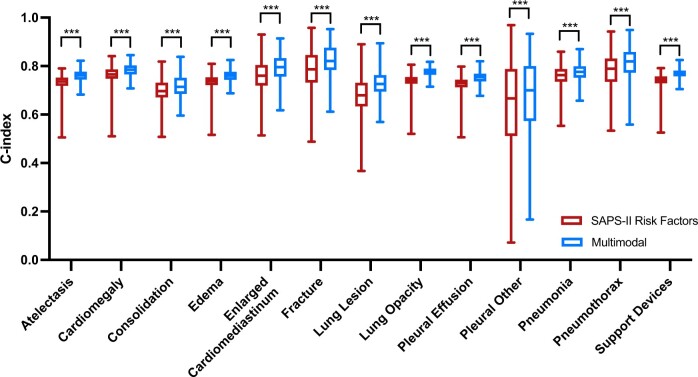
The C-index results of the models trained on the entire dataset and tested on the patients with different chest X-ray abnormalities. ***P < .001.

Figure 4 shows the C-index results of our SAPS-II risk factors and multimodal features models, marked in red and blue, respectively. Both are trained on the entire dataset and tested on patients with normal or abnormal chest X-rays. Our multimodal features model outperforms the SAPS-II risk factors model, and our model can more accurately predict normal subjects. Figure 5 further breaks chest X-ray abnormalities into 13 predefined thorax diseases.

### Comparison of different types of text features

We compare the results of our model using different types of text features. Simplified Acute Physiology Score-II risk factors + labels, SAPS-II risk factors + transformer features, and SAPS-II risk factors + GCN features. They are trained using 15 routine physiological measurements combined with 14 thorax disease labels, transformer-based features, and GCN-based features, respectively. Table 2 lists the results of our model using these 3 feature settings. The mean C-indexes for these 3 settings are 0.7669 (0.7456-0.7882), 0.7714 (0.7488-0.7941), and 0.7720 (0.7517-0.7923), respectively. Models with transformer or GCN features outperform models that only use labels. However, there is no significant difference between the transformer and GCN features. These findings are important as they demonstrate that incorporating advanced feature extraction methods, such as transformer and GCN, can improve model performance compared to traditional labels alone. Moreover, the lack of a significant difference between transformer and GCN features suggests that both methods are equally viable for enhancing predictive accuracy in this context. This study adds to the growing body of evidence supporting the integration of advanced feature extraction techniques in predictive modeling, providing a basis for further exploration and optimization in future research.

**Table 2.** The C-index results of the models using different types of text features.

Model	C-index (95% CI)
SAPS-II risk factors + labels	0.7669 (0.7456-0.7882)
SAPS-II risk factors + transformer features	0.7714 (0.7488-0.7941)
SAPS-II risk factors + GCN features	0.7720 (0.7517-0.7923)

Abbreviations: GCN, graph convolutional network; SAPS-II, Simplified Acute Physiology Score II.

### Contribution of thorax diseases in survival analysis

Next, we analyze the multivariate association of chest X-ray abnormalities to ICU mortality based on Cox Proportion Hazards (CoxPH model) (Table 3). The *P*-values of these 4 findings, enlarged cardiomediastinum, fracture, pneumonia, and pneumothorax, are greater than .05, indicating no statistically significant difference. In other words, these findings do not contribute to mortality prediction. It highlights the importance of using a comprehensive set of clinical and radiological features in predictive modeling. While individual chest X-ray abnormalities may not be significant predictors, their inclusion in a broader context of physiological measurements and other clinical data can enhance the overall predictive accuracy. Our findings contribute to the ongoing discussion in the literature about the relative importance of various features in ICU mortality prediction and suggest avenues for future research to explore combinations of features that may yield more significant predictive power.

**Table 3.** Multivariate associations of chest X-ray abnormalities to ICU mortality.

Abnormality	Hazard ratio	95% CI	*P*
Atelectasis	0.84	0.75-0.94	^a^
Cardiomegaly	0.85	0.76-0.96	^a^
Consolidation	1.33	1.14-1.55	^b^
Edema	1.23	1.10-1.38	^b^
Enlarged cardiomediastinum	0.91	0.75-1.12	.37
Fracture	0.96	0.72-1.28	.77
Lung lesion	1.37	1.13-1.67	^a^
Lung opacity	1.29	1.17-1.42	^b^
Pleural effusion	1.13	1.02-1.26	^c^
Pleural other	0.64	0.41-1.00	^c^
Pneumonia	1.07	0.93-1.23	.34
Pneumothorax	1.10	0.86-1.41	.45
Support devices	1.27	1.16-1.39	^b^

Abbreviation: ICU, intensive care unit.

a *P* ≤ .01.

b *P* < .001.

c *P* ≤ .05.

### Comparison of linear and deep survival models

We then compare the performances of the linear machine learning and deep learning models: CoxPH^38^ and DeepSurv-based model. Table 4 shows the results for both models with 2 feature settings. The average C-indexes of the CoxPH model with SAPS-II risk factors and SAPS-II risk factors + labels are 0.7510 (0.7300-0.7720) and 0.7617 (0.7414-0.7819), respectively, in comparison with 0.7545 (0.7240-0.7849) and 0.7669 (0.7456-0.7882) obtained by our DeepSurv-based model. The results demonstrate that deep learning models outperform CoxPH on high-dimensional features. The *P*-value for the CoxPH and DeepSurv-based model using SAPS-II is .01, and the *P*-value is 1.08e-6 when using SAPS-II + labels.

**Table 4.** The C-index results of the linear machine learning models and the deep learning models trained and tested on the entire dataset.

Model		C-index (95% CI)
SAPS-II risk factors	CoxPH	0.7510 (0.7300-0.7720)
	DeepSurv-based	**0.7545 (0.7240-0.7849)**
SAPS-II risk factors + labels	CoxPH	0.7617 (0.7414-0.7819)
	DeepSurv-based	**0.7669 (0.7456-0.7882)**

Performance metrics across various models on the test set. Bold values indicate the best-performing metric for each category.

Abbreviation: SAPS-II, Simplified Acute Physiology Score II.

These findings are significant as they highlight the advantages of deep learning models in handling high-dimensional data. They offer superior predictive performance compared to traditional linear models like CoxPH. The significant *P*-values indicate that the differences in performance are statistically meaningful, underscoring the robustness of the DeepSurv-based model.

### Error analysis

**Figure 6.**
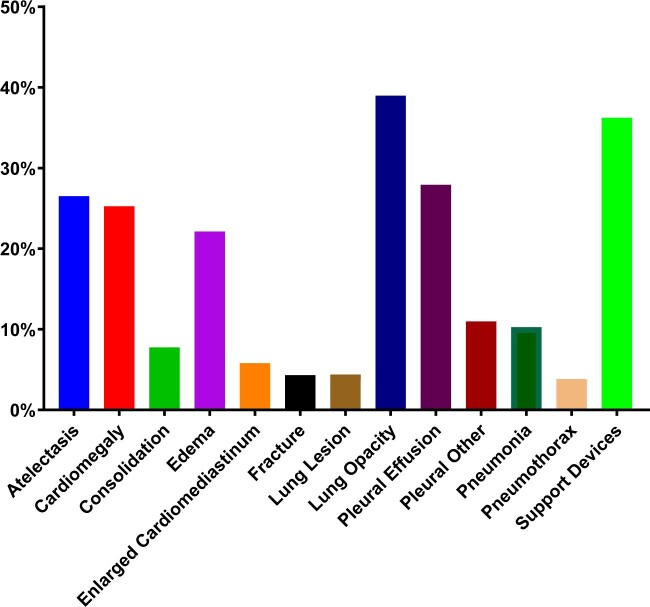
Distributions of thorax diseases among patients where our multimodal model made more accurate predictions than SAPS-II. Abbreviation: SAPS-II, Simplified Acute Physiology Score II.

Error analysis (ie, examining the reasons behind inaccurate predictions) revealed that the multimodal accounted for fewer errors. Table S3 demonstrates one example case of ICU mortality. According to physiological measurements, SAPS-II graded patient #1 with a score of 38 and patient #2 with 36. However, patient #1 was decreased at hour 198, but patient #2 was deceased at hour 75. Hence, the SAPS-II incorrectly assigned the score. However, our multimodal approach correctly assigned a higher survival probability to patient #1 (0.9903) than to patient #2 (0.9562). In one bootstrap sample, we observed a total of 40 529 such errors (patient #1 has a normal chest X-ray, and SAPS-II gives wrong predictions, but our multimodal method gives correct predictions) with 1802 distinct patients, out of which 527 patients have normal chest X-rays and 1275 patients have abnormal chest X-rays. Figure 6 shows the distribution of thorax diseases among 1275 patients. It shows that lung opacity (38.98%) contributes most to the ICU mortality prediction.

## Discussion

Our study demonstrates the potential of integrating multimodal data, including structured clinical data, radiology reports, and chest X-ray images, into deep learning models to improve ICU mortality prediction. By enriching traditional ICU scoring systems with additional textual and imaging features, we observed a significant improvement in predictive accuracy. Specifically, our multimodal features model achieved an average C-index of 0.7829, outperforming the baseline SAPS-II scoring model.

A key finding in our study is the benefit of incorporating advanced feature extraction methods, such as BERT-based text representations and GCN-based features, into the prediction model. These techniques enabled our model to capture more nuanced information from unstructured radiology reports, contributing to the overall improvement in performance. We also demonstrated the efficacy of early average fusion, showing that multimodal feature integration can yield better predictive accuracy than unimodal models.

Our results also highlight the limitations of relying solely on traditional ICU scoring systems. While the SAPS-II score provides a solid baseline, excluding unstructured data, such as radiology reports and images, limits its predictive power. Adding these features allows for a more comprehensive assessment of patient risk, as demonstrated by the superior performance of our multimodal model.

There are several limitations to this work. First, we use a fusion strategy similar to “early fusion” to fuse the text and image features extracted by BlueBERT and ChexNet, respectively, but their parameters are not updated during the training iterations. In the future, we plan to use joint fusion to propagate the loss back to the feature extraction modules during training, which may improve the representation learning performance. Second, a knowledge graph is a popular tool for representing background knowledge, which can improve several aspects of the model. We will explore other domain knowledge and try different ways of incorporating the knowledge graph into ICU mortality prediction. Third, the longitudinal EHR data contain information regarding the disease progressions that may help ICU mortality prediction but are not utilized in this work. In the future, we can employ the longitudinal EHR to assist in predicting ICU mortality. To account for long and irregular intervals between consecutive longitudinal multimodal data points, we suggest modifying traditional positional encoding to embed visit times directly into high-dimensional representations.^39^ This adaptation allows us to incorporate information about visit times by performing an elementwise addition of time step embeddings to the embeddings of multimodal data. Fourth, there is a risk of selection bias in this study. For instance, our analysis only included patients with imaging studies after ICU admission. For example, imaging studies are usually performed to confirm central line placement when a patient is sicker. This selection could lead to a sample not representative of the ICU population. However, selection bias is a common problem in machine learning,^40^ statistics,^41^ and epidemiology^42^; as a result, several techniques have been developed to correct it. In the future, we will investigate these techniques. Fifth, machine learning models are vulnerable to adversarial attacks.^43^ For example, images can be attacked by adding a small perturbation to the original images. Texts can be attacked by adding a small number of words. These attacks are imperceptible to humans but mislead a model into producing incorrect outputs. Like selection bias, adversarial attack is a common problem in the medical domain, where accurate diagnostic results are paramount.^44^ Previous studies suggest that if a model could eliminate noises in their learned feature representations, they would be more robust against adversarial perturbations.^45^ We will study these techniques to improve the robustness of the model in the future. To enhance trustworthy artificial intelligence (AI) development on ICU mortality prediction, we can also incorporate interpretability into our framework.^46^ Sixth, as large language models (LLMs) have shown their power in NLP, LLMs can be considered for text feature extraction in the future.

While our work only scratches the surface of multimodal fusion for survival analysis, we hope it will shed light on the future directions for ICU mortality prediction.

## Supplementary Material

ooae137_Supplementary_Data

## Data Availability

We made our codes publicly available at https://github.com/bionlplab/mimic-icu-mortality. The dataset we use in this work is Medical Information Mart for Intensive Care IV (MIMIC-IV), also publicly available at https://physionet.org/content/mimiciv/0.4/.

## References

[1] Lipshutz AK , Feiner JR, Grimes B, Gropper MA. Predicting mortality in the intensive care unit: a comparison of the University Health Consortium expected probability of mortality and the Mortality Prediction Model III. J Intensive Care. 2016;4:35-38. 27217959 10.1186/s40560-016-0158-z PMC4876564

[2] Zimmerman JE , Kramer AA, McNair DS, Malila FM. Acute physiology and chronic health evaluation (APACHE) IV: hospital mortality assessment for today’s critically ill patients. Crit Care Med. 2006;34:1297-1310. 16540951 10.1097/01.CCM.0000215112.84523.F0

[3] Le Gall J-R , Lemeshow S, Saulnier F. A new simplified acute physiology score (SAPS II) based on a European/North American multicenter study. JAMA. 1993;270:2957-2963. 8254858 10.1001/jama.270.24.2957

[4] Teres D , Lemeshow S, Avrunin JS, Pastides H. Validation of the mortality prediction model for ICU patients. Crit Care Med. 1987;15:208-213. 3816253 10.1097/00003246-198703000-00005

[5] El-Rashidy N , El-Sappagh S, Abuhmed T, Abdelrazek S, El-Bakry HM. Intensive care unit mortality prediction: an improved patient-specific stacking ensemble model. IEEE Access. 2020;8:133541-133564.

[6] Ghassemi M , Pimentel MA, Naumann T, et al. A multivariate timeseries modeling approach to severity of illness assessment and forecasting in ICU with sparse, heterogeneous clinical data. In: *Proceedings of the AAAI Conference on Artificial Intelligence*, 2015 Jan. 2015:446-453. PMC4864016 27182460

[7] Zhao Z , Chen A, Hou W, et al Prediction model and risk scores of ICU admission and mortality in COVID-19. PLoS One. 2020;15:e0236618. 32730358 10.1371/journal.pone.0236618 PMC7392248

[8] Johnson AEW , Mark RG. Real-time mortality prediction in the Intensive Care Unit. In: *AMIA Annual Symposium Proceedings*. American Medical Informatics Association, 2018 Apr 16. 2017:994-1003. PMC5977709 29854167

[9] Murdoch TB , Detsky AS. The inevitable application of big data to health care. JAMA. 2013;309:1351-1352. 23549579 10.1001/jama.2013.393

[10] Ching T , Himmelstein DS, Beaulieu-Jones BK, et al Opportunities and obstacles for deep learning in biology and medicine. J R Soc Interface. 2018;15:20170387. 29618526 10.1098/rsif.2017.0387 PMC5938574

[11] Mechcatie E , Rosenberg K. Nursing notes are predictive of outcomes in ICU patients. Am J Nurs. 2018;118:70. 10.1097/01.NAJ.0000546385.96797.29 30260891

[12] Yang H , Kuang L, Xia F. Multimodal temporal-clinical note network for mortality prediction. J Biomed Semantics. 2021;12:3-14. 33588949 10.1186/s13326-021-00235-3 PMC7885612

[13] Grnarova P , Schmidt F, Hyland SL, Eickhoff C. Neural document embeddings for intensive care patient mortality prediction. arXiv, arXiv:1612.00467, 2016 Dec 1, preprint: not peer reviewed.

[14] Huang S-C , Pareek A, Seyyedi S, Banerjee I, Lungren MP. Fusion of medical imaging and electronic health records using deep learning: a systematic review and implementation guidelines. NPJ Digit Med. 2020;3:136-139. 33083571 10.1038/s41746-020-00341-z PMC7567861

[15] Liu N , Wang K, Jin X, Gao B, Dellandréa E, Chen L. Visual affective classification by combining visual and text features. PLoS One. 2017;12:e0183018. 28850566 10.1371/journal.pone.0183018 PMC5574549

[16] Liu J , Chen Y, Lan L, et al Prediction of rupture risk in anterior communicating artery aneurysms with a feed-forward artificial neural network. Eur Radiol. 2018;28:3268-3275. 29476219 10.1007/s00330-017-5300-3

[17] Liu M-Q. Bone age assessment model based on multi-dimensional feature fusion using deep learning. Acad J Second Mil Med Univ. 2018;12:909-916.

[18] Bakkali S , Ming Z, Coustaty M, Rusiñol M. Visual and textual deep feature fusion for document image classification. In: *Proceedings of the IEEE/CVF Conference on Computer Vision and Pattern Recognition Workshops*, virtual. 2020:562-563.

[19] Reda I , Khalil A, Elmogy M, et al Deep learning role in early diagnosis of prostate cancer. Technol Cancer Res Treat. 2018;17:1533034618775530. 29804518 10.1177/1533034618775530 PMC5972199

[20] Qiu S , Chang GH, Panagia M, Gopal DM, Au R, Kolachalama VB. Fusion of deep learning models of MRI scans, Mini–Mental State Examination, and logical memory test enhances diagnosis of mild cognitive impairment. Alzheimers Dement (Amst). 2018;10:737-749. 30480079 10.1016/j.dadm.2018.08.013 PMC6240705

[21] Yala A , Lehman C, Schuster T, Portnoi T, Barzilay R. A deep learning mammography-based model for improved breast cancer risk prediction. Radiology. 2019;292:60-66. 31063083 10.1148/radiol.2019182716

[22] Kawahara J , Daneshvar S, Argenziano G, Hamarneh G. Seven-point checklist and skin lesion classification using multitask multimodal neural nets. IEEE J Biomed Health Inform. 2019;23:538-546. 10.1109/JBHI.2018.2824327 29993994

[23] Yoo Y , Tang LY, Li DK, et al Deep learning of brain lesion patterns and user-defined clinical and MRI features for predicting conversion to multiple sclerosis from clinically isolated syndrome. Comput Methods Biomechan Biomed Eng Imaging Vis. 2019;7:250-259.

[24] Ford E , Carroll JA, Smith HE, Scott D, Cassell JA. Extracting information from the text of electronic medical records to improve case detection: a systematic review. J Am Med Inform Assoc. 2016;23:1007-1015. 26911811 10.1093/jamia/ocv180 PMC4997034

[25] Weissman GE , Hubbard RA, Ungar LH, et al Inclusion of unstructured clinical text improves early prediction of death or prolonged ICU stay. Crit Care Med. 2018;46:1125-1132. 29629986 10.1097/CCM.0000000000003148 PMC6005735

[26] Johnson A , Bulgarelli L, Pollard T, Horng S, Celi LA, Mark IVR. Mimic-iv (version 0.4). PhysioNet. Accessed November 26, 2024. https://physionet.org/content/mimiciv/0.4/

[27] Cox DR. Regression models and life‐tables. J R Stat Soc Ser B Methodol. 1972;34:187-202.

[28] Katzman JL , Shaham U, Cloninger A, Bates J, Jiang T, Kluger Y. DeepSurv: personalized treatment recommender system using a Cox proportional hazards deep neural network. BMC Med Res Methodol. 2018;18:24-12. 29482517 10.1186/s12874-018-0482-1 PMC5828433

[29] Wang X , Peng Y, Lu L, Lu Z, Bagheri M, Summers RM. ChestX-ray8: hospital-scale chest X-ray database and benchmarks on weakly-supervised classification and localization of common thorax diseases. In: *Proceedings of the IEEE Conference on Computer Vision and Pattern Recognition*, Honolulu, US. 2017:2097-2106.

[30] Irvin J , Rajpurkar P, Ko M, et al Chexpert: a large chest radiograph dataset with uncertainty labels and expert comparison. In: *Proceedings of the AAAI Conference on Artificial Intelligence*, Honolulu, US. 2019;33:590-597.

[31] Johnson AE , Pollard TJ, Greenbaum NR, et al MIMIC-CXR-JPG, a large publicly available database of labeled chest radiographs. arXiv, arXiv:1901.07042, 2019 Jan 21, preprint: not peer reviewed.

[32] Peng Y , Wang X, Lu L, Bagheri M, Summers R, Lu Z. NegBio: a high-performance tool for negation and uncertainty detection in radiology reports. AMIA Jt Summits Transl Sci Proc. 2018;2018:188. PMC5961822 29888070

[33] Kipf TN , Welling M. Semi-supervised classification with graph convolutional networks. arXiv. arXiv:1609.02907, preprint: not peer reviewed. 2016 Sep 9.

[34] Huang G , Liu Z, Van Der Maaten L, Weinberger KQ. Densely connected convolutional networks. In: *Proceedings of the IEEE Conference on Computer Vision and Pattern Recognition*, Honolulu, US. 2017:4700-4708.

[35] Rajpurkar P , Irvin J, Zhu K, et al Chexnet: radiologist-level pneumonia detection on chest X-rays with deep learning. arXiv, arXiv:1711.05225, 2017, preprint: not peer reviewed.

[36] Kingma DP , Ba J. Adam: a method for stochastic optimization. arXiv, arXiv:1412.6980, 2014, preprint: peer reviewed.

[37] Peng Y , Yan S, Lu Z. Transfer learning in biomedical natural language processing: an evaluation of BERT and ELMo on ten benchmarking datasets. In: *Proceedings of the 18th BioNLP Workshop and Shared Task*, 2019 Aug. 2019:58-65.

[38] Pölsterl S. scikit-survival: a library for time-to-event analysis built on top of scikit-learn. J Mach Learn Res. 2020;21:1-6.34305477

[39] Holste G , Lin M, Zhou R, et al Harnessing the power of longitudinal medical imaging for eye disease prognosis using Transformer-based sequence modeling. *NPJ Digit Med*. 2024;7:216. 10.1038/s41746-024-01207-4 PMC11329720 39152209

[40] Cortes C , Mohri M, Riley M, Rostamizadeh A. Sample selection bias correction theory. In: *International Conference on Algorithmic Learning Theory*, Berlin, Heidelberg. Springer; 2008:38-53.

[41] Whittemore AS. Collapsibility of multidimensional contingency tables. J R Stat Soc Ser B Methodol. 1978;40:328-340.

[42] Robins JM. Data, design, and background knowledge in etiologic inference. Epidemiology. 2001;12:313-320. 11338312 10.1097/00001648-200105000-00011

[43] Goodfellow IJ , Shlens J, Szegedy C. Explaining and harnessing adversarial examples. arXiv, arXiv:1412.6572, 2014 Dec 20, preprint: not peer reviewed.

[44] Paschali M , Conjeti S, Navarro F, Navab N. Generalizability vs robustness: investigating medical imaging networks using adversarial examples. In: *International Conference on Medical Image Computing and Computer-Assisted Intervention*, Granada, Spain. Springer; 2018:493-501.

[45] Finlayson SG , Bowers JD, Ito J, Zittrain JL, Beam AL, Kohane IS. Adversarial attacks on medical machine learning. Science. 2019;363:1287-1289. 30898923 10.1126/science.aaw4399 PMC7657648

[46] Hou B , Li H, Jiao Z, Zhou Z, Zheng H, Fan Y. Deep clustering survival machines with interpretable expert distributions. Proc IEEE Int Symp Biomed Imaging. 2023;2023:1-4. 10.1109/isbi53787.2023.10230844 PMC10544788 37790880

