# Supplementary material for: An empirical study of using radiology reports and images to improve intensive care unit mortality prediction
Source: JAMIA Open. 2025 Feb 20;8(1):ooae137. doi: 10.1093/jamiaopen/ooae137 (PMC11841685; doi:10.1093/jamiaopen/ooae137)
Supplement: ooae137_Supplementary_Data [file ooae137_supplementary_data.docx]

**Supplementary Table 1**. Information of the ICU admission group. EC: Enlarged Cardiomediastinum.

|  | **ICU Discharge** | **ICU mortality** |
| --- | --- | --- |
| Patient, n | 7,715 | 2,213 |
| Age, mean (SD), y | 61.78 (18.20) | 69.63 (14.84) |
| Gender, male/female, % | 45/55 | 46/54 |
| Common thorax diseases, % |  |  |
| Atelectasis | 19.90 | 21.69 |
| Cardiomegaly | 17.64 | 18.08 |
| Consolidation | 4.19 | 8.00 |
| Edema | 13.45 | 19.52 |
| EC | 3.54 | 4.07 |
| Fracture | 2.68 | 2.21 |
| Lung Lesion | 2.64 | 4.97 |
| Lung Opacity | 26.36 | 36.69 |
| Pleural Effusion | 17.04 | 28.29 |
| Pleural Other | 0.62 | 0.72 |
| Pneumonia | 0.70 | 9.31 |
| Pneumothorax | 2.44 | 2.94 |
| Support Devices | 34.50 | 43.83 |

**Supplementary Table 2**. SAPS-II physiological measurements of the ICU admission group.

|  | **Score** | **ICU Discharge %** | **ICU mortality %** |
| --- | --- | --- | --- |
| Age, year |  |  |  |
| <40 | 0 | 12.51 | 3.21 |
| 40-59 | 7 | 25.91 | 17.67 |
| 60-69 | 12 | 21.63 | 20.70 |
| 70-74 | 15 | 9.16 | 11.84 |
| 75-79 | 16 | 9.19 | 11.21 |
| >=80 | 18 | 21.59 | 35.38 |
| Heart Rate |  |  |  |
| <40 | 11 | 21.04 | 31.22 |
| 40-69 | 2 | 42.28 | 28.11 |
| 70-119 | 0 | 34.71 | 32.90 |
| 120-159 | 4 | 0.93 | 2.94 |
| >=160 | 7 | 1.04 | 4.84 |
| Systolic BP, mmHg |  |  |  |
| <70 | 13 | 5.51 | 22.01 |
| 70-99 | 5 | 62.49 | 59.51 |
| 100-199 | 0 | 30.82 | 17.44 |
| >=200 | 2 | 1.18 | 1.04 |
| Temperature <= 39 ^O^C |  |  |  |
| No | 0 | 95.58 | 93.76 |
| Yes | 3 | 4.42 | 6.24 |
| PaO_2_/FiO_2_, mmHg |  |  |  |
| <100 | 11 | 3.47 | 13.29 |
| 100-199 | 9 | 7.57 | 13.92 |
| >=200 | 6 | 13.66 | 17.40 |
| No ventilation | 0 | 75.29 | 55.40 |
| Blood urea nitrogen, mg/dL |  |  |  |
| <28 | 0 | 70.28 | 47.40 |
| 28-93 | 6 | 26.80 | 46.18 |
| >=84 | 10 | 2.92 | 6.42 |
| Urine output, mL/day |  |  |  |
| <500 | 11 | 7.66 | 29.60 |
| 500-999 | 4 | 16.84 | 19.84 |
| >=1000 | 0 | 75.50 | 50.56 |
| Sodium, mEq/L |  |  |  |
| <125 | 5 | 2.20 | 2.58 |
| 125-144 | 0 | 89.31 | 81.16 |
| >=145 | 1 | 8.49 | 16.27 |
| Potassium |  |  |  |
| 3.0-4.9 | 0 | 82.31 | 69.95 |
| <3.0 or >=5.0 | 3 | 17.69 | 30.05 |
| Bicarbonate, mEq/L |  |  |  |
| <15 | 6 | 4.72 | 16.49 |
| 15-19 | 3 | 18.09 | 26.25 |
| >=20 | 0 | 77.19 | 67.25 |
| Bilirubim, mg/dL |  |  |  |
| <4.0 | 0 | 96.64 | 90.24 |
| 4.0-5.9 | 4 | 1.24 | 2.76 |
| >=6.0 | 9 | 2.11 | 7.00 |
| White blood count, x10^3^/mm^3^ |  |  |  |
| <1.0 | 12 | 0.45 | 2.08 |
| 1.0-19.9 | 0 | 89.38 | 77.09 |
| >=20.0 | 3 | 10.16 | 20.83 |
| Glasgow coma scale |  |  |  |
| 14-15 | 0 | 78.70 | 68.59 |
| 11-13 | 5 | 11.67 | 10.94 |
| 9-10 | 7 | 3.69 | 9.47 |
| 6-8 | 13 | 3.85 | 7.14 |
| <6 | 26 | 2.09 | 7.86 |
| Chronic disease |  |  |  |
| None | 0 | 90.56 | 76.59 |
| Metastatic cancer | 9 | 6.13 | 15.68 |
| Hematologic malignancy | 10 | 2.40 | 7.05 |
| AIDS | 17 | 0.91 | 0.68 |
| Type of admission |  |  |  |
| Scheduled surgical | 0 | 0.75 | 0.09 |
| Medical | 6 | 83.50 | 86.58 |
| Unscheduled surgical | 8 | 15.75 | 13.33 |

**Supplementary Table 3**. n example case of ICU mortality prediction where our multimodal model made more accurate prediction than SAPS-II. According to physiological measurements, SAPS-II graded patient 1 the score of 38 and patient 2 36. However, patient 1 was decreased at hour 198, but patient 2 was deceased at hour 75. Hence, the SAPS-II incorrectly assigned the score. However, our multimodal approach correctly assigned a higher survival probability to patient 1 (0.9903) to patient 2 (0.9562).

|  | **Patient #1** | **Patient #2** |
| --- | --- | --- |
| Age, y | 85 | 62 |
| Gender | Male | Male |
| Race | African American | White |
| Time-to-event, hr | 198 | 75 |
| Mortality score | 38 | 36 |
| Findings | No Findings | Atelectasis, Consolidation, Lung Lesion, Pleural Effusion |
| Report | FINDINGS: Portable AP chest radiograph demonstrates no focal consolidation, pleural effusion, pulmonary vascular engorgement, or pneumothorax. Multiple vascular stents are noted in the right upper extremity, superior mediastinum, and mid left upper extremity. The aorta is tortuous. The cardiomediastinal silhouette is otherwise normal.  IMPRESSION: No acute cardiopulmonary process. | FINDINGS: There continues to be near-complete opacification of the right lung compatible with components of pleural effusion and post-obstructive consolidation secondary to a known right chest mass. Additionally, there is increasing left pleural effusion with basilar atelectasis. Assessment of the heart size is limited due to these opacities. There is no pneumothorax.  IMPRESSION: Stable appearance of right-sided pleural effusion and post-obstructive consolidation in the setting of a known right chest mass; increasing left pleural effusion with basal atelectasis. |
| Chest X-ray | 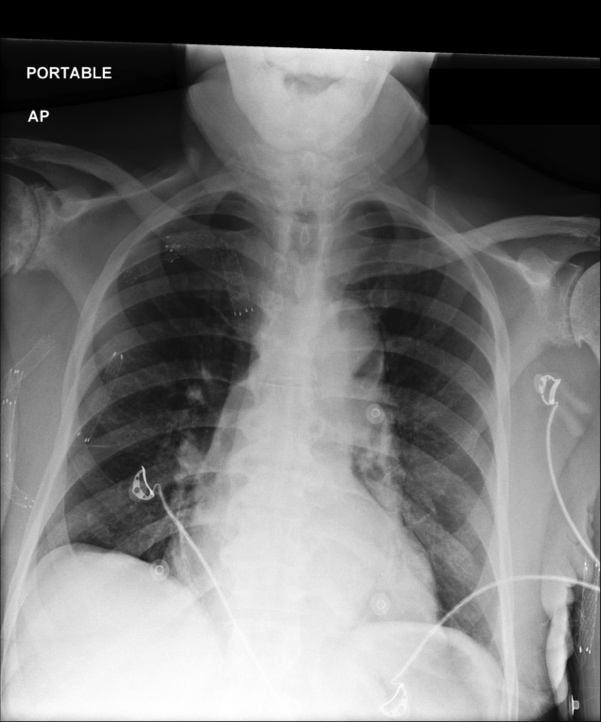 | 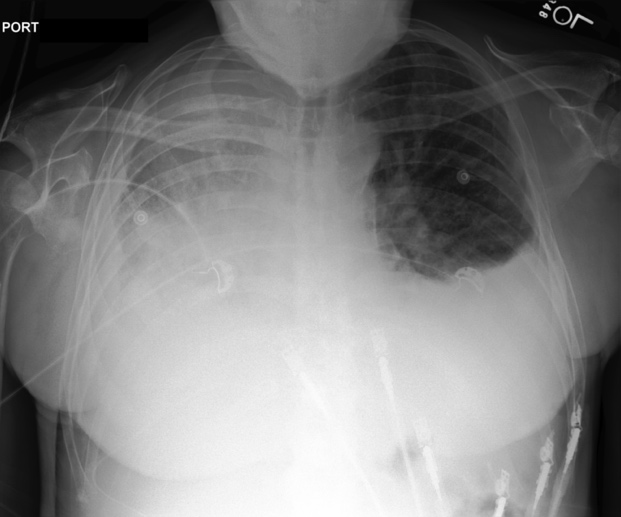 |
